# (+)-(1*S*,5*R*,6*R*)-6-[(*S*)-1-Hy­droxy-2-(meth­oxy­meth­yloxy)eth­yl]-1-methyl-3-trichloro­methyl-2-aza-4,7-dioxa­bicyclo­[3.3.0]oct-2-en-8-one

**DOI:** 10.1107/S1600536812042912

**Published:** 2012-10-20

**Authors:** Takeshi Oishi, Hiroki Oishi, Syun Tsuzaki, Takaaki Sato, Noritaka Chida

**Affiliations:** a School of Medicine, Keio University, Hiyoshi 4-1-1, Kohoku-ku, Yokohama 223-8521, Japan; b Department of Applied Chemistry, Faculty of Science and Technology, Keio University, Hiyoshi 3-14-1, Kohoku-ku, Yokohama 223-8522, Japan

## Abstract

In the title compound, C_11_H_14_Cl_3_NO_6_, the fused five-membered oxazoline and tetra­hydro­furan rings are essentially planar with maximum deviations of 0.069 (1) and 0.031 (1) Å, respectively, and make a dihedral angle of 64.23 (11)° with each other. In the crystal, mol­ecules are linked by O—H⋯O and C—H⋯O hydrogen bonds, forming chains along the *b*-axis direction. Further C—H⋯O hydrogen bonds are observed between the chains.

## Related literature

For the synthesis, see: Oishi *et al.* (2012 ▶). For the isolation of sphingofungins, see: VanMiddlesworth, Giacobbe *et al.* (1992 ▶); VanMiddlesworth, Dufresne *et al.* (1992 ▶); Horn *et al.* (1992 ▶).
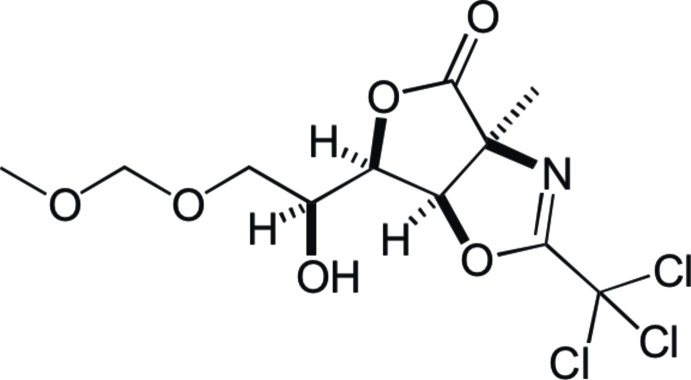


## Experimental

### 

#### Crystal data

C_11_H_14_Cl_3_NO_6_
*M* *_r_* = 362.58
Monoclinic, 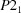

*a* = 8.9311 (7) Å
*b* = 6.0283 (4) Å
*c* = 13.8694 (10) Å
β = 99.699 (2)°
*V* = 736.05 (9) Å^3^
*Z* = 2
Mo *K*α radiation
μ = 0.65 mm^−1^
*T* = 90 K
0.50 × 0.25 × 0.16 mm

#### Data collection

Bruker D8 goniometer diffractometer
Absorption correction: multi-scan (*SADABS*; Bruker, 2012 ▶) *T*_min_ = 0.738, *T*_max_ = 0.904
6662 measured reflections
2364 independent reflections
2291 reflections with *I* > 2σ(*I*)
*R*_int_ = 0.028

#### Refinement

*R*[*F* ^2^ > 2σ(*F* ^2^)] = 0.027
*wR*(*F* ^2^) = 0.069
*S* = 1.34
2364 reflections
193 parameters
1 restraint
H-atom parameters constrained
Δρ_max_ = 0.37 e Å^−3^
Δρ_min_ = −0.22 e Å^−3^
Absolute structure: Flack (1983 ▶), 947 Friedel pairs
Flack parameter: 0.01 (5)

### 

Data collection: *APEX2* (Bruker, 2012 ▶); cell refinement: *SAINT* (Bruker, 2012 ▶); data reduction: *SAINT*; program(s) used to solve structure: *SHELXS97* (Sheldrick, 2008 ▶); program(s) used to refine structure: *SHELXL97* (Sheldrick, 2008 ▶); molecular graphics: *Mercury* (Macrae *et al.*, 2006 ▶); software used to prepare material for publication: *SHELXL97*.

## Supplementary Material

Crystal structure: contains datablock(s) global, I. DOI: 10.1107/S1600536812042912/is5207sup1.cif

Structure factors: contains datablock(s) I. DOI: 10.1107/S1600536812042912/is5207Isup2.hkl

Supplementary material file. DOI: 10.1107/S1600536812042912/is5207Isup3.cdx

Supplementary material file. DOI: 10.1107/S1600536812042912/is5207Isup4.cml

Additional supplementary materials:  crystallographic information; 3D view; checkCIF report

## Figures and Tables

**Table 1:** Hydrogen-bond geometry (Å, °)

*D*—H⋯*A*	*D*—H	H⋯*A*	*D*⋯*A*	*D*—H⋯*A*
O12—H12⋯O15^i^	0.84	1.90	2.695 (2)	157
C6—H6⋯O9^ii^	1.00	2.43	3.402 (3)	164
C17—H17*C*⋯O9^iii^	0.98	2.53	3.320 (3)	137

Symmetry codes: (i) 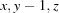; (ii) 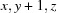; (iii) 
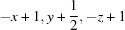
.

**Fig. 1.**
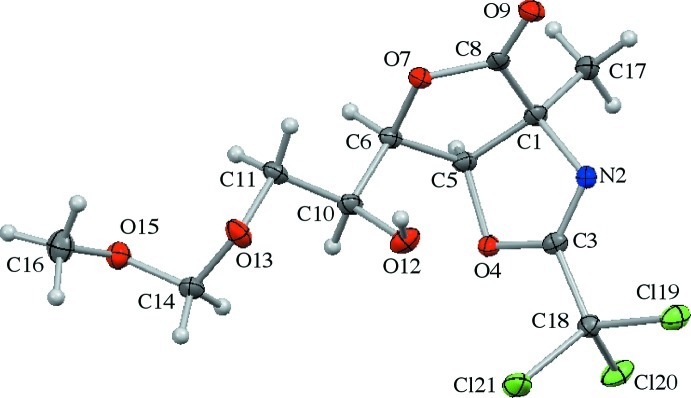
The molecular structure of the title compound, with atom labels and 50% probability displacement ellipsoids for non-H atoms.

**Fig. 2.**
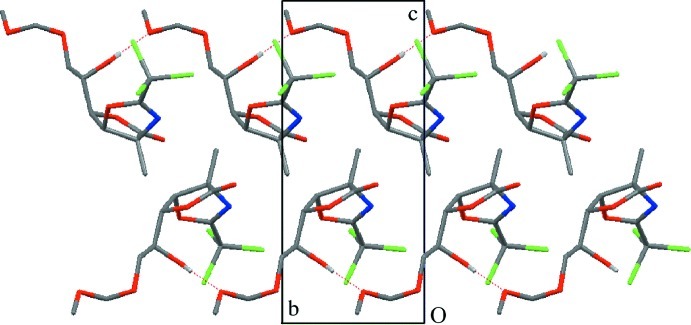
The crystal packing of the title compound, viewed down the a axis. The dashed lines indicate O—H···O hydrogen bonds.

## supplementary crystallographic information

### Comment

Sphingofungins are natural antifungal agents isolated from *Aspergillus* and reported to be potent inhibitors of the biosynthesis of sphingolipids (VanMiddlesworth, Giacobbe *et al.*, 1992; VanMiddlesworth, Dufresne *et al.*, 1992; Horn *et al.*, 1992). The title compound (I), C_11_H_14_Cl_3_NO_6_, which has four contiguous stereogenic center including a tetra-substituted carbon with nitrogen (Fig. 1), was provided in a synthetic study on the natural products sphingofungins from _D_-ribose (Oishi *et al.*, 2012). The absolute configurations were confirmed by the X-ray analysis as C1*S*, C5*R*, C6*R* and C10*S*. The crystal packing was stabilized by an intermolecular O—H···O hydrogen bond, forming molecular a chain along the *b* axis (Fig. 2). There are also C—H···O hydrogen bonds and intermolecular Cl···O short contacts, Cl19···O4 (*x*, *y* - 1, *z*) and Cl20···O7 (*x* + 1, *y*, *z*) being 3.070 (2) and 3.142 (2) Å, respectively.

### Experimental

The title compound was obtained in a synthetic study of sphingofungins from _D_-ribose (Oishi *et al.*, 2012), and recrystallized from ethyl acetate solution. [α]^27^_D_ +83.1 (*c* 0.345, CHCl_3_); m.p. 409.7–411.2 K.

### Refinement

C-bound H atoms were positioned geometrically with C—H = 0.98–1.00 Å, and constrained to ride on their parent atoms with *U*_iso_(H) = 1.2*U*_eq_(C) or 1.5*U*_eq_(methyl C). The H atom of hydroxyl group (O12) was placed guided by difference maps, with O—H = 0.84 Å and with *U*_iso_(H) = 1.5*U*_eq_(O). Three reflections (6 3 6, 6 3 7, 0 1 16) have been omitted in the final refinement.

### Crystal data

**Table:**

C_11_H_14_Cl_3_NO_6_	*D*_x_ = 1.636 Mg m^−^^3^
*M**_r_* = 362.58	Melting point: 409.7 K
Monoclinic, *P*2_1_	Mo *K*α radiation, λ = 0.71073 Å
*a* = 8.9311 (7) Å	Cell parameters from 5778 reflections
*b* = 6.0283 (4) Å	θ = 2.3–25.1°
*c* = 13.8694 (10) Å	µ = 0.65 mm^−^^1^
β = 99.699 (2)°	*T* = 90 K
*V* = 736.05 (9) Å^3^	Prism, colourless
*Z* = 2	0.50 × 0.25 × 0.16 mm
*F*(000) = 372	

### Data collection

**Table:**

Bruker D8 goniometer diffractometer	2364 independent reflections
Radiation source: fine-focus sealed tube	2291 reflections with *I* > 2σ(*I*)
Graphite monochromator	*R*_int_ = 0.028
Detector resolution: 10.4167 pixels mm^-1^	θ_max_ = 25.1°, θ_min_ = 2.3°
ω scans	*h* = −10→10
Absorption correction: multi-scan (*SADABS*; Bruker, 2012)	*k* = −7→6
*T*_min_ = 0.738, *T*_max_ = 0.904	*l* = −16→16
6662 measured reflections	

### Refinement

**Table:**

Refinement on *F*^2^	Secondary atom site location: difference Fourier map
Least-squares matrix: full	Hydrogen site location: inferred from neighbouring sites
*R*[*F*^2^ > 2σ(*F*^2^)] = 0.027	H-atom parameters constrained
*wR*(*F*^2^) = 0.069	*w* = 1/[σ^2^(*F*_o_^2^) + (0.0338*P*)^2^] where *P* = (*F*_o_^2^ + 2*F*_c_^2^)/3
*S* = 1.34	(Δ/σ)_max_ = 0.001
2364 reflections	Δρ_max_ = 0.37 e Å^−^^3^
193 parameters	Δρ_min_ = −0.22 e Å^−^^3^
1 restraint	Absolute structure: Flack (1983), 947 Friedel pairs
Primary atom site location: structure-invariant direct methods	Flack parameter: 0.01 (5)

### Special details

**Table:**

Geometry. All e.s.d.'s (except the e.s.d. in the dihedral angle between two l.s. planes) are estimated using the full covariance matrix. The cell e.s.d.'s are taken into account individually in the estimation of e.s.d.'s in distances, angles and torsion angles; correlations between e.s.d.'s in cell parameters are only used when they are defined by crystal symmetry. An approximate (isotropic) treatment of cell e.s.d.'s is used for estimating e.s.d.'s involving l.s. planes.
Refinement. Refinement of *F*^2^ against ALL reflections. The weighted *R*-factor *wR* and goodness of fit *S* are based on *F*^2^, conventional *R*-factors *R* are based on *F*, with *F* set to zero for negative *F*^2^. The threshold expression of *F*^2^ > σ(*F*^2^) is used only for calculating *R*-factors(gt) *etc*. and is not relevant to the choice of reflections for refinement. *R*-factors based on *F*^2^ are statistically about twice as large as those based on *F*, and *R*- factors based on ALL data will be even larger.

### Fractional atomic coordinates and isotropic or equivalent isotropic displacement parameters (Å^2^)

**Table:**

	*x*	*y*	*z*	*U*_iso_*/*U*_eq_	
C1	0.7195 (2)	0.5236 (4)	0.43062 (15)	0.0152 (5)	
N2	0.7869 (2)	0.3750 (3)	0.36335 (12)	0.0139 (4)	
C3	0.8554 (2)	0.5035 (4)	0.31378 (15)	0.0134 (5)	
O4	0.85179 (17)	0.7255 (3)	0.32714 (10)	0.0156 (3)	
C5	0.7464 (2)	0.7590 (4)	0.39563 (15)	0.0148 (5)	
H5	0.7928	0.8550	0.4516	0.018*	
C6	0.5893 (2)	0.8481 (4)	0.34831 (15)	0.0142 (5)	
H6	0.5652	0.9825	0.3851	0.017*	
O7	0.48036 (16)	0.6720 (3)	0.36046 (10)	0.0162 (4)	
C8	0.5464 (2)	0.4965 (4)	0.40903 (15)	0.0142 (5)	
O9	0.47385 (18)	0.3412 (3)	0.42994 (10)	0.0181 (4)	
C10	0.5688 (3)	0.9041 (4)	0.24039 (15)	0.0161 (5)	
H10	0.6464	1.0185	0.2316	0.019*	
C11	0.4125 (3)	1.0049 (4)	0.20566 (15)	0.0165 (5)	
H11A	0.3335	0.9027	0.2216	0.020*	
H11B	0.4039	1.1462	0.2408	0.020*	
O12	0.59596 (18)	0.7153 (3)	0.18539 (11)	0.0208 (4)	
H12	0.5132	0.6520	0.1642	0.031*	
O13	0.38660 (19)	1.0460 (3)	0.10212 (11)	0.0205 (4)	
C14	0.4558 (3)	1.2396 (4)	0.07599 (16)	0.0192 (5)	
H14A	0.4577	1.2365	0.0049	0.023*	
H14B	0.5622	1.2437	0.1105	0.023*	
O15	0.38097 (18)	1.4326 (3)	0.09848 (11)	0.0195 (4)	
C16	0.2416 (3)	1.4735 (5)	0.03432 (17)	0.0256 (6)	
H16A	0.1663	1.3625	0.0456	0.038*	
H16B	0.2582	1.4641	−0.0336	0.038*	
H16C	0.2045	1.6220	0.0468	0.038*	
C17	0.7852 (3)	0.4676 (4)	0.53576 (15)	0.0194 (5)	
H17A	0.8962	0.4793	0.5454	0.029*	
H17B	0.7564	0.3159	0.5503	0.029*	
H17C	0.7455	0.5714	0.5796	0.029*	
C18	0.9561 (2)	0.4317 (4)	0.24145 (15)	0.0155 (5)	
Cl19	0.95353 (6)	0.14171 (10)	0.22820 (4)	0.02283 (16)	
Cl20	1.14459 (6)	0.51691 (10)	0.28730 (4)	0.02415 (16)	
Cl21	0.89393 (7)	0.55359 (12)	0.12613 (4)	0.03251 (19)	

### Atomic displacement parameters (Å^2^)

**Table:**

	*U*^11^	*U*^22^	*U*^33^	*U*^12^	*U*^13^	*U*^23^
C1	0.0208 (12)	0.0143 (12)	0.0113 (10)	0.0026 (11)	0.0052 (9)	−0.0013 (10)
N2	0.0157 (10)	0.0130 (10)	0.0135 (8)	0.0008 (8)	0.0040 (8)	0.0008 (8)
C3	0.0127 (11)	0.0159 (12)	0.0107 (10)	−0.0001 (10)	−0.0005 (8)	−0.0026 (10)
O4	0.0157 (8)	0.0130 (8)	0.0195 (8)	−0.0007 (7)	0.0071 (6)	0.0001 (7)
C5	0.0158 (11)	0.0172 (13)	0.0122 (10)	0.0006 (10)	0.0050 (8)	−0.0015 (11)
C6	0.0191 (12)	0.0100 (11)	0.0147 (10)	−0.0041 (10)	0.0063 (9)	−0.0026 (10)
O7	0.0162 (8)	0.0161 (9)	0.0174 (8)	−0.0006 (7)	0.0061 (6)	0.0032 (7)
C8	0.0219 (12)	0.0125 (12)	0.0094 (10)	0.0023 (11)	0.0065 (9)	−0.0024 (10)
O9	0.0228 (9)	0.0154 (9)	0.0174 (7)	−0.0033 (7)	0.0074 (7)	0.0001 (7)
C10	0.0195 (12)	0.0159 (12)	0.0143 (10)	−0.0009 (10)	0.0064 (9)	−0.0022 (10)
C11	0.0216 (11)	0.0175 (13)	0.0109 (10)	−0.0012 (10)	0.0041 (9)	0.0026 (10)
O12	0.0208 (9)	0.0239 (9)	0.0179 (8)	0.0014 (8)	0.0035 (7)	−0.0072 (8)
O13	0.0294 (9)	0.0177 (9)	0.0135 (8)	−0.0011 (8)	0.0010 (7)	0.0023 (7)
C14	0.0231 (13)	0.0195 (12)	0.0164 (11)	0.0038 (11)	0.0071 (9)	0.0027 (11)
O15	0.0219 (9)	0.0171 (8)	0.0194 (8)	0.0012 (7)	0.0038 (7)	0.0004 (8)
C16	0.0234 (13)	0.0262 (15)	0.0257 (12)	0.0023 (11)	−0.0005 (10)	−0.0014 (12)
C17	0.0190 (12)	0.0231 (14)	0.0168 (11)	0.0052 (10)	0.0048 (9)	0.0037 (11)
C18	0.0158 (12)	0.0159 (12)	0.0151 (11)	−0.0009 (10)	0.0032 (9)	−0.0003 (10)
Cl19	0.0254 (3)	0.0168 (3)	0.0290 (3)	−0.0034 (3)	0.0126 (3)	−0.0074 (3)
Cl20	0.0162 (3)	0.0259 (3)	0.0317 (3)	−0.0044 (3)	0.0079 (2)	−0.0094 (3)
Cl21	0.0345 (4)	0.0461 (5)	0.0202 (3)	0.0192 (3)	0.0139 (3)	0.0130 (3)

### Geometric parameters (Å, º)

**Table:**

C1—N2	1.491 (3)	C11—H11A	0.9900
C1—C17	1.515 (3)	C11—H11B	0.9900
C1—C5	1.532 (3)	O12—H12	0.8400
C1—C8	1.533 (3)	O13—C14	1.396 (3)
N2—C3	1.260 (3)	C14—O15	1.403 (3)
C3—O4	1.352 (3)	C14—H14A	0.9900
C3—C18	1.519 (3)	C14—H14B	0.9900
O4—C5	1.459 (3)	O15—C16	1.424 (3)
C5—C6	1.542 (3)	C16—H16A	0.9800
C5—H5	1.0000	C16—H16B	0.9800
C6—O7	1.468 (3)	C16—H16C	0.9800
C6—C10	1.515 (3)	C17—H17A	0.9800
C6—H6	1.0000	C17—H17B	0.9800
O7—C8	1.337 (3)	C17—H17C	0.9800
C8—O9	1.202 (3)	C18—Cl19	1.757 (3)
C10—O12	1.414 (3)	C18—Cl21	1.763 (2)
C10—C11	1.524 (3)	C18—Cl20	1.773 (2)
C10—H10	1.0000	Cl19—O4^i^	3.0697 (17)
C11—O13	1.437 (2)	Cl20—O7^ii^	3.1419 (16)
			
N2—C1—C17	109.67 (18)	O13—C11—H11A	109.3
N2—C1—C5	104.89 (16)	C10—C11—H11A	109.3
C17—C1—C5	117.1 (2)	O13—C11—H11B	109.3
N2—C1—C8	108.29 (17)	C10—C11—H11B	109.3
C17—C1—C8	112.22 (18)	H11A—C11—H11B	107.9
C5—C1—C8	104.10 (18)	C10—O12—H12	109.5
C3—N2—C1	104.83 (19)	C14—O13—C11	113.55 (17)
N2—C3—O4	120.6 (2)	O13—C14—O15	112.79 (17)
N2—C3—C18	125.5 (2)	O13—C14—H14A	109.0
O4—C3—C18	113.8 (2)	O15—C14—H14A	109.0
C3—O4—C5	105.01 (17)	O13—C14—H14B	109.0
O4—C5—C1	103.34 (17)	O15—C14—H14B	109.0
O4—C5—C6	114.30 (16)	H14A—C14—H14B	107.8
C1—C5—C6	106.11 (18)	C14—O15—C16	113.79 (18)
O4—C5—H5	110.9	O15—C16—H16A	109.5
C1—C5—H5	110.9	O15—C16—H16B	109.5
C6—C5—H5	110.9	H16A—C16—H16B	109.5
O7—C6—C10	107.59 (17)	O15—C16—H16C	109.5
O7—C6—C5	105.81 (18)	H16A—C16—H16C	109.5
C10—C6—C5	116.62 (18)	H16B—C16—H16C	109.5
O7—C6—H6	108.9	C1—C17—H17A	109.5
C10—C6—H6	108.9	C1—C17—H17B	109.5
C5—C6—H6	108.9	H17A—C17—H17B	109.5
C8—O7—C6	112.65 (16)	C1—C17—H17C	109.5
O9—C8—O7	121.9 (2)	H17A—C17—H17C	109.5
O9—C8—C1	127.1 (2)	H17B—C17—H17C	109.5
O7—C8—C1	111.04 (19)	C3—C18—Cl19	110.75 (17)
O12—C10—C11	112.46 (18)	C3—C18—Cl21	110.46 (16)
O12—C10—C6	110.51 (19)	Cl19—C18—Cl21	108.87 (12)
C11—C10—C6	110.68 (17)	C3—C18—Cl20	108.21 (15)
O12—C10—H10	107.7	Cl19—C18—Cl20	108.69 (13)
C11—C10—H10	107.7	Cl21—C18—Cl20	109.84 (12)
C6—C10—H10	107.7	C18—Cl19—O4^i^	139.97 (8)
O13—C11—C10	111.67 (17)	C18—Cl20—O7^ii^	177.84 (8)
			
C17—C1—N2—C3	−118.3 (2)	C17—C1—C8—O9	−47.7 (3)
C5—C1—N2—C3	8.3 (2)	C5—C1—C8—O9	−175.3 (2)
C8—C1—N2—C3	119.0 (2)	N2—C1—C8—O7	−105.6 (2)
C1—N2—C3—O4	−1.7 (3)	C17—C1—C8—O7	133.2 (2)
C1—N2—C3—C18	173.61 (19)	C5—C1—C8—O7	5.6 (2)
N2—C3—O4—C5	−6.0 (3)	O7—C6—C10—O12	−59.9 (2)
C18—C3—O4—C5	178.18 (16)	C5—C6—C10—O12	58.7 (3)
C3—O4—C5—C1	10.3 (2)	O7—C6—C10—C11	65.4 (2)
C3—O4—C5—C6	−104.5 (2)	C5—C6—C10—C11	−176.0 (2)
N2—C1—C5—O4	−11.3 (2)	O12—C10—C11—O13	−52.1 (3)
C17—C1—C5—O4	110.49 (19)	C6—C10—C11—O13	−176.20 (19)
C8—C1—C5—O4	−125.02 (17)	C10—C11—O13—C14	−79.6 (2)
N2—C1—C5—C6	109.24 (18)	C11—O13—C14—O15	−74.1 (2)
C17—C1—C5—C6	−128.9 (2)	O13—C14—O15—C16	−74.3 (2)
C8—C1—C5—C6	−4.4 (2)	N2—C3—C18—Cl19	4.6 (3)
O4—C5—C6—O7	115.31 (19)	O4—C3—C18—Cl19	−179.78 (15)
C1—C5—C6—O7	2.1 (2)	N2—C3—C18—Cl21	125.3 (2)
O4—C5—C6—C10	−4.2 (3)	O4—C3—C18—Cl21	−59.1 (2)
C1—C5—C6—C10	−117.4 (2)	N2—C3—C18—Cl20	−114.4 (2)
C10—C6—O7—C8	126.83 (19)	O4—C3—C18—Cl20	61.2 (2)
C5—C6—O7—C8	1.5 (2)	C3—C18—Cl19—O4^i^	−12.9 (2)
C6—O7—C8—O9	176.28 (19)	Cl21—C18—Cl19—O4^i^	−134.58 (9)
C6—O7—C8—C1	−4.6 (2)	Cl20—C18—Cl19—O4^i^	105.81 (13)
N2—C1—C8—O9	73.5 (3)		

Symmetry codes: (i) *x*, *y*−1, *z*; (ii) *x*+1, *y*, *z*.

### Hydrogen-bond geometry (Å, º)

**Table:**

*D*—H···*A*	*D*—H	H···*A*	*D*···*A*	*D*—H···*A*
O12—H12···O15^i^	0.84	1.90	2.695 (2)	157
C6—H6···O9^iii^	1.00	2.43	3.402 (3)	164
C17—H17*C*···O9^iv^	0.98	2.53	3.320 (3)	137

Symmetry codes: (i) *x*, *y*−1, *z*; (iii) *x*, *y*+1, *z*; (iv) −*x*+1, *y*+1/2, −*z*+1.
